# Mesodermal Progenitor Cells (MPCs) Differentiate into Mesenchymal Stromal Cells (MSCs) by Activation of Wnt5/Calmodulin Signalling Pathway

**DOI:** 10.1371/journal.pone.0025600

**Published:** 2011-09-29

**Authors:** Rita Fazzi, Simone Pacini, Vittoria Carnicelli, Luisa Trombi, Marina Montali, Edoardo Lazzarini, Mario Petrini

**Affiliations:** 1 Hematology Division, Department of Oncology, Transplants and New Advances in Medicine, University of Pisa, Pisa, Italy; 2 Dipartimento di Scienze dell'Uomo e dell'Ambiente, University of Pisa, Pisa, Italy; University of Minnesota, United States of America

## Abstract

**Background:**

Mesenchymal Stromal Cells (MSCs) remain poorly characterized because of the absence of manifest physical, phenotypic, and functional properties in cultured cell populations. Despite considerable research on MSCs and their clinical application, the biology of these cells is not fully clarified and data on signalling activation during mesenchymal differentiation and proliferation are controversial. The role of Wnt pathways is still debated, partly due to culture heterogeneity and methodological inconsistencies. Recently, we described a new bone marrow cell population isolated from MSC cultures that we named Mesodermal Progenitor Cells (MPCs) for their mesenchymal and endothelial differentiation potential. An optimized culture method allowed the isolation from human adult bone marrow of a highly pure population of MPCs (more than 97%), that showed the distinctive SSEA-4^+^CD105^+^CD90^neg^ phenotype and not expressing MSCA-1 antigen. Under these selective culture conditions the percentage of MSCs (SSEA-4^neg^CD105^+^CD90^bright^ and MSCA-1^+^), in the primary cultures, resulted lower than 2%.

**Methodology/Principal Finding:**

We demonstrate that MPCs differentiate to MSCs through an SSEA-4^+^CD105^+^CD90^bright^ early intermediate precursor. Differentiation paralleled the activation of Wnt5/Calmodulin signalling by autocrine/paracrine intense secretion of Wnt5a and Wnt5b (*p*<0.05 vs uncondictioned media), which was later silenced in late MSCs (SSEA-4^neg^). We found the inhibition of this pathway by calmidazolium chloride specifically blocked mesenchymal induction (ID_50_ = 0.5 µM, *p*<0.01), while endothelial differentiation was unaffected.

**Conclusion:**

The present study describes two different putative progenitors (*early* and *late* MSCs) that, together with already described MPCs, could be co-isolated and expanded in different percentages depending on the culture conditions. These results suggest that some modifications to the widely accepted MSC nomenclature are required.

## Introduction

The Wnt family of signaling proteins participate in multiple developmental events during embryogenesis [1], [2], [3]. In addition, it has also been implicated in adult tissue homeostasis [4], [5]. Wnt signals are pleiotropic, with effects that include mitogenic stimulation, cell fate specification, and differentiation [6]. Wnts are highly conserved, cysteine-rich secreted ligands which bind the Frizzled (Fzd) receptor family. So far, 19 Wnts have been identified in humans together with 10 Fzd receptors, co-receptors (LRP-5, LRP-6), and inhibitors (Dkks, sFrps and Wif). Binding of Wnt ligands to the Fzds results in the activation of different pathways: the *canonical* pathway that involves nuclearization and activation of β-catenin [7], and the β-catenin independent *non-canonical* pathways acting through phosphokinase networks [8], including JNKs, CaMK-II, PKC, and Calmodulin/NF-AT.

The role of Wnt signalling in Mesenchymal Stromal Cell (MSC) fate is still debated. Both *canonical* and *non-canonical* pathways have been implicated in mesenchymal differentiation and proliferation. This “dual” role could be related to the specific Wnt ligand responsible for the signalling and/or to the developmental stage at the time of Wnt pathway engagement (reviewed by Ling L. *et al.* [9]). In cultured MSCs, the *canonical* Wnt3a activated signalling seems to stimulate proliferation and self-renewal [10], whereas the *non-canonical* Wnt5a/JNK mediated signalling inhibits proliferation and promotes osteogenic differentiation [11]. Some authors have reported that *non-canonical* Wnt signalling inhibits MSC proliferation in either autocrine or paracrine fashion [12]. Moreover, ligand concentration in the culture medium could lead to opposite effects [13]. Thus, the role of Wnt signalling in mesenchymal fate is far from clarified and the controversial results could rise from intrinsic variability and heterogeneity of the MSC preparations.

MSCs are still poorly characterized due to the absence of manifest physical, phenotypic, and functional properties in heterogeneous cell culture populations [14], which contain single stem cell-like cells as well as progenitor cells with different lineage commitments [15]. Also, cell origin and culture conditions induce high variability in cell composition that can affect interpretation of the experimental results [16]. A further obstacle to the study of MSCs is the lack of precise knowledge about their *in vivo* identity. Once established in culture, they express a variety of cell-lineage specific antigens, including adhesion molecules, integrins, and growth factor receptors that are either down- or up-regulated in MSC (sub)populations [17]. Moreover, the number of culture passages required to select a homogenous MSC population may induce loss of immature and multipotent precursor cells.

A number of markers have been proven to be suitable for the prospective isolation of MSCs from primary tissues. They include CD271, SSEA-4, ganglioside GD2, CD146, CD200, and the α_v_β_5_ integrin complex, as well as several antibody-defined molecules [18], [19], [20], [21], [22]. However, none of them represents an ultimate and exclusive MSC marker.

Recently, we identified in MSC cultures a novel cell population characterized by unusual morphology and a unique phenotype [23]. These cells exhibited both mesenchymal and endothelial differentiation potential and therefore we named them Mesodermal Progenitor Cells (MPCs). We optimized a protocol to harvest MPCs from human Bone Marrow Mono-Nucleate Cells (BMMNCs) supplemented with autologous serum [24]. They consisted of a highly homogeneous population identified by phenotype CD105^+^SSEA-4^+^CD90^neg^ while lacking many other mesenchymal associated markers, including MSCA-1, CD166, CD271, W5B5 [24], as well as the pericyte marker CD146. MPCs revealed the expression of the pluripotency-associated marker SSEA-4 and of nuclear factors *Oct-4* and *Nanog* [25]. We also demonstrated that different serum supplementations in MSC culture medium led to different percentages of co-cultured MPCs [23], thus contributing to the variability of cell proliferation and differentiation potential [26]. Interestingly, when cultured in appropriate conditions MPCs differentiated to either highly proliferative and clonogenic MSCs or to mature endothelial cells showing tube-like structures in Matrigel® 3D-cultures.

Here we demonstrate that MPCs differentiate to MSCs through an SSEA-4^+^ early intermediate precursor. Differentiation was paralleled by the activation of the *non-canonical* Wnt5/Calmodulin signalling pathway. The specificity of the pathway, subsequently silenced during differentiation into mature multipotent SSEA-4^neg^ MSCs, was confirmed by culturing MPCs in the presence of inhibitors. The inhibition of *non-canonical* Wnt5/Calmodulin signalling impaired MSC differentiation leaving endothelial induction unaffected.

## Materials and Methods

### Ethical statement

The study protocol was approved by the ethical committee of the Azienda Ospedaliera Universitaria Pisana. The fundamental principles of ethics in research on human participants were maintained throughout the study period according to the principles expressed in the Declaration of Helsinki. The research procedures were disclosed to all participants and written informed consent was obtained for sample collection.

### Primary cell cultures

Bone marrow samples were obtained from 4 patients (2M/2F, median age 69 years) undergoing cardiac surgery. MSC cultures were obtained from bone marrow mononuclear cells (BMMNCs) grown under standard conditions, using DMEM (Invitrogen, Carlsband CA-USA) supplemented with 10% FBS (Invitrogen). MPCs were isolated from BMMNCs cultured in DMEM supplemented with 10% pooled human AB serum, obtained from male donors only (PhABS, Lonza, Walkersville MD-USA), as previously described [23]. Media were changed every 48 h and cultures maintained at 37°C and 5% CO_2_ for 10–12 days, then detached by TrypLE Select® (Invitrogen) digestion and processed for characterization and mRNA extraction.

### Cytofluorimetric characterization and validation of primary cultures

Aliquots of the detached cells were washed in PBS/0.5% BSA and stained with anti-SSEA-4 AlexaFluor 488-conjugated (Biolegend), anti-MSCA-1 PE-conjugated (Miltenyi Biotec, Gladbach GER), and anti-CD90 PE/Cy5-conjugated (BectonDickinson, San Jose CA-SA). Samples were acquired using FACSCanto II® (BectonDickinson) and analyzed by Diva Software®. A mesenchymal component (SSEA-4^neg^MSCA-1^+^CD90^+^) lower than 2% was the cut off point for MPCs samples to be selected for further analysis.

### Molecular characterization and Wnt signalling qPCRArray

Total RNA was extracted using RNeasy Mini Kit (Qiagen GmbH, Hilden GER) as indicated by the manufacturer's protocol. On-column DNase I digestion was performed. 100 ng RNA samples were retrotranscribed with QuantiTect® Whole Transcriptome Kit (Qiagen). 50-fold cDNA dilutions were analyzed by quantitative Real Time PCR, using an iCycler-iQ5 Optical System (Bio-Rad Laboratories, Hercules, CA-USA) and iQ SYBR Green SuperMix (Bio-Rad). All samples were run in duplicate. Primers for OCT4, NANOG, SOX15, SOX9, NESTIN, SPP1, FBX15, and RUNX2 genes were obtained as previously described [24]. Relative quantitative analysis was carried out following the 2^−ΔΔCt^ Livak method [27]. GAPDH and HPRT housekeeping genes were used for normalization. Wnt related genes expression profile analysis was performed using Wnt signalling pathway RT^2^ Profiler™ PCR array kit from SABioscience (Quiagen) according to manufacturer's instructions. Data were analyzed by SABioscience web-base PCR Array Data Analysis tool and expressed as 2^−ΔΔCt^. Gene expression was defined “*consistent*” for values over 0.01, “*mild*” for values between 0.01 and 0.001, while genes were considered “*not expressed*” for values lower than 0.001.

### MPC mesenchymal differentiation and Slot-Blot analysis

Five bone marrow samples (3M/2F, median age 64) were cultured in PhABS to isolate MPCs, as described above. After cytofluorimetrical validation, cells were detached and plated (10'000 cells/cm^2^) in MesenPRO™ RS (Invitrogen) tissue culture treated (TC) 6-well plates, to induce mesenchymal differentiation.

After 7 days (T1) conditioned medium was collected, high speed centrifuged and processed for Slot-Blot analysis. The procedure was repeated after further 7 days (T2).

T1 and T2 conditioned media were microfiltrated in quadruplicate, using Bio-Dot® Microfiltration Apparatus (BioRad, Hercules CA-USA) and blotted onto nitrocellulose membranes. Membranes were processed to evaluate secreted proteins using anti-Wnt5a, anti-Wnt5b, and anti-Dkk1 (all antibodies from ABCam, Cambridge UK). Briefly, membranes were blocked in 0.05% Tween20–5% BSA TBS, incubated for 2 hs with 1 µg/ml purified primary antibody, washed three times in 0.05% Tween20 TBS and then incubated with HRP conjugated secondary antibody (ABCam) for 1 h. After three washings, membranes were incubated with chemoluminescent ECL reagent (ABCam) and images immediately acquired by ChemiDoc digital imaging system (Biorad). Densitometric evaluations were performed using Leica QWin image analysis software (Leica, Wetzlar Germany). Data were presented as *Net Pixel Density*, calculated by subtracting median grey levels of bands. T1 and T2 cultures were also processed for anti-SSEA-4 and CD90 immunofluorescence staining and citofluorimetric characterization for CD105, CD90, and SSEA-4 expression.

### Inhibition of MPC mesenchymal differentiation

Mesenchymal differentiation was inhibited by culturing MPCs in the presence of anti-Wnt5a antibody, anti-Wnt5b antibody (ABCam), or a combination of anti-Wnt5a/anti-Wnt5b at 0.5 µg/ml and 2.0 µg/ml respectively. IgG isotype antibody (BectonDickinson) was used as a control. In parallel, inhibitors of downstream phosphokinases in the *non-canonical Ca^2+^-dependent* signalling pathways were used. In detail, PKC and CaMK-II were inhibited by adding different doses (5 µl, 10 µl and 50 µl) of the inhibitor cocktail from Millipore (Billerica, MA-USA). Autocamtide-2-related inhibitory peptide (AIP) at 0.5 µM, 3.0 µM, and 5.0 µM concentrations, or calmidazolium chloride (CLMDZ) at 0.5 µM, 1.0 µM, and 2.0 µM concentrations were also used. Differentiation was measured by percentage of reduced AlamarBlue (%ABred). At day 7 of culture 10% v/v AlamarBlue® (Invitrogen) was added to the culture medium and 6 hs after treatment 100 µl samples were photometrically assayed at 570 nm/600 nm, following manufacturer's instructions.

### Inhibition of Calmudulin activity in MPC-derived MSC expansion

MPCs from five bone marrow samples (3M/2F, median age 66) were induced to differentiate into MSCs by culturing in MesenPRO™ RS for 7 days in duplicate (T1). MSCs were cultured for further 7 days (T2) in the presence (0.5 µM and 1.0 µM) or absence of CLMDZ and proliferation was assayed. Cells from untreated cultures were detached and replated at 5,000 cells/cm^2^ with or without CLMDZ. After 7 days of culture (T3) the AlamarBlue® assay was performed. Data were expressed as *Inhibition Index* calculated according to the formula:
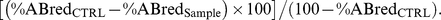



### Inhibition of Ca^2+^-dependent signalling pathways by CLMDZ in MPC endothelial differentiation

Detached MPCs were induced to differentiate toward the endothelial lineage as previously reported [24], [25], in the presence (1.0 µM and 2.0 µM) and absence of CLMDZ. Briefly, MPCs were plated at a density of 10,000 cells/cm^2^ in fibronectin coated 12-well plates (BectonDickinson) and cultured for 7 days in EndoCult® medium (StemCell, Vancouver Canada). Evaluation of differentiation and the consequent pre-endothelial cell proliferation was performed using AlamarBlue® assay, as reported above. After pre-differentiation, cells were detached by trypsin digestion (Invitrogen) and 50,000 cells seeded on Matrigel™ (BectonDickinson) to perform the tube-like formation assay as previously described [25]. After 24 h of culture in EGM-2 medium (Lonza) supplemented with 50 ng/ml VEGF, phase contrast images of the tube-like network were acquired and 30–50 capillary-like tubes per sample were measured using Qwin software.

### Statistical analysis

Statistical analysis was performed using two tailed *t-student* test. In alternative, for non-parametric series of data, Mann-Whitney test was also performed, *p*<0.05 was considered to be significant.

## Results

**Figure 1:**
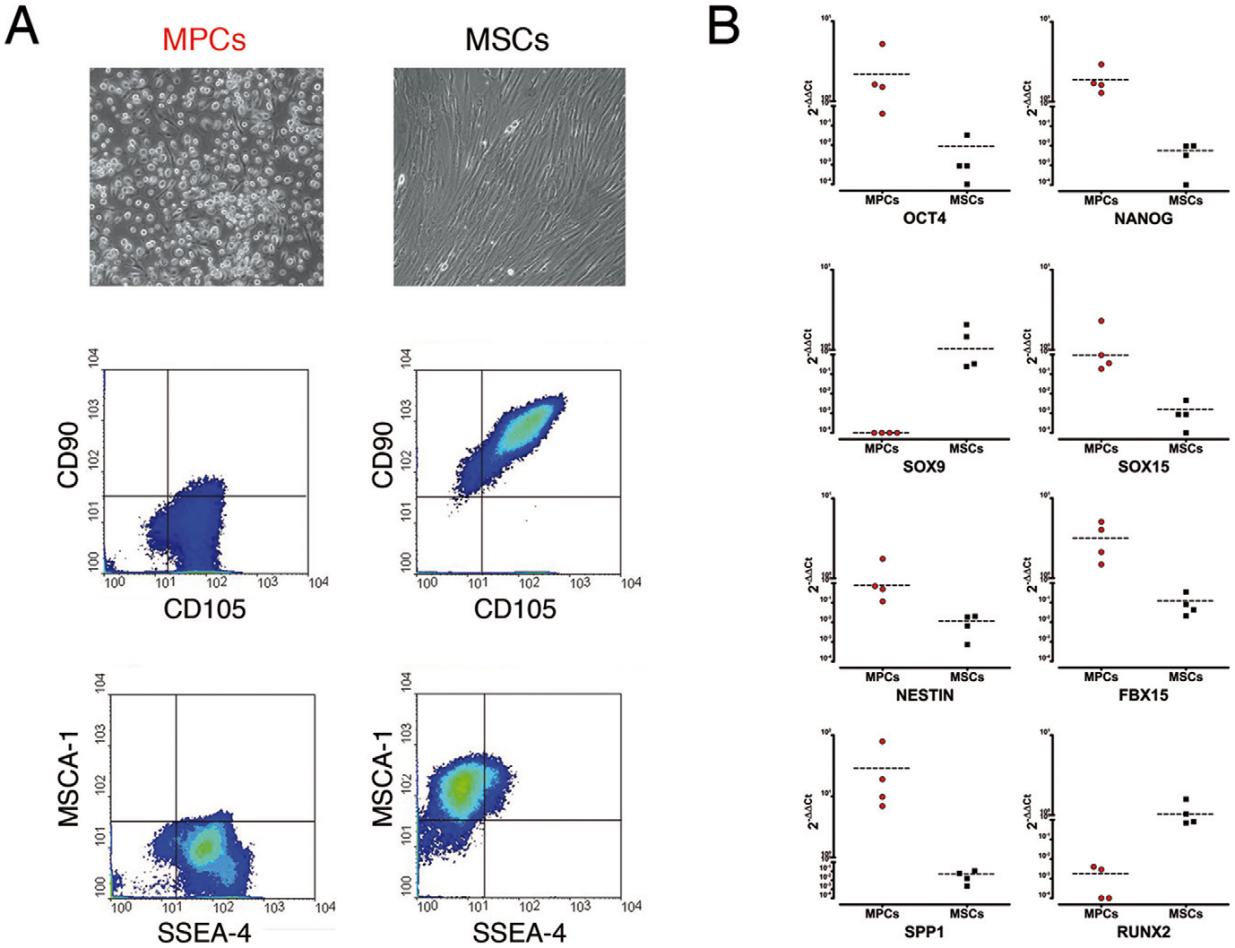
Characterization of MPC and MSC primary cultures. Culturing BMMNCs in PhABS or FBS resulted in two highly monomorphic cultures of MPCs and MSCs, respectively. (A) MPCs were rounded and highly rifrangent (Leica DM-IRB 100X, phase contrast) expressing SSEA-4, low levels of CD105, and no CD90 and MSCA-1 MSC markers. Cytofluorimetric analysis showed high purity for both cultures. (B) Gene expression profiling confirmed the peculiar molecular signature of MCPs (red dots) characterized by the expression of pluripotency-associated genes. MSC (black squares) profile was significantly different from that of MPC (*p*<0.01), with the high expression of RUNX2 and SOX9.

We used two culture settings (PhABS vs FBS) that allowed us to specifically separate MPCs from MSCs in primary cultures from whole bone marrow samples. Under those conditions over 98% purity for either population was obtained (Figure 1A). Molecular characterization revealed a distinctive profile for MPCs characterized by the expression of functional pluripotency-associated genes, including OCT4 (isoform A) and NANOG (Figure 1B). SOX15, NESTIN, FBX15 and SPP1, this latest reported as functional Oct-4 homodimer target genes [28], were also expressed at significant levels (*p*<0.01, Figure 1B) confirming the activation of the peculiar adult Oct-4 circuit, previously reported [25]. MSCs expressed mesenchymal associated genes, including SOX9 and RUNX2 while lacking pluripotency markers (Figure 1B).

**Figure 2:**
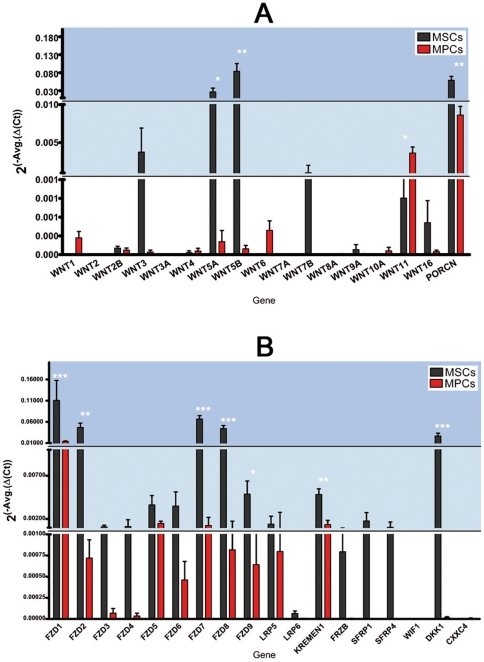
Quantitative RT-PCR assay for Wnts, Fzd receptors, co-receptors and inhibitors. (A) No Wnts mRNA were detected in MPCs (red bars) except for WNT11 that showed *mild* expression. MSCs (black bars) showed *consistent* expression of WNT5A and WNT5B and *mild* expression of WNT3 and WNT7B. PORCN was expressed in both populations. (B) MPCs showed *consistent* expression of FZD1 only, while MSCs expressed a number of FZD receptors and Wnt signalling inhibitors. (* *p*<0.05, ** *p*<0.01, *** *p*<0.001).

RT^2^ Profile™ PCR arrays revealed 16 different Wnt mRNAs (Figure 2A). MPCs showed *mild* expression (0.001<2^−ΔΔCt^≤0.01) of WNT11 only that was not expressed in MSCs (*p*<0.05). MSCs showed *consistent* expression (2^−ΔΔCt^>0.01) of WNT5A and WNT5B and *mild* expression of WNT3 and WNT7B. Porcupine homolog *Drosophila* gene (PORCN) was expressed at different levels in both MPCs and MSCs (*p*<0.01), suggesting that translated Wnt proteins are processed for secretion. Surface receptor Fzd profiles (Figure 2B) were characterized by *consistent* expression of FZD1 in both MPCs and MSCs. However, MSCs expressed 10 times higher levels of the FZD1 transcript as compared to MPCs (*p*<0.001) and revealed positive amplification, at different levels, for any of the other FZD receptors investigated. Interestingly, we detected transcripts for LRP-5 and KREMEN1 co-receptors both in MSCs and MPCs, but only MSCs showed *consistent* expression of dickkopf homolog 1 (DKK1). Our data suggest that Wnt signalling in MPCs was allowed by the expression of FZD1, which binds at high affinity the *canonical* pathway effectors Wnt3a and β_1_-catenin as well as the *non-canonical* ligands Wnt5a, Wnt5b, and Wnt7b [29] (Table S1).

**Figure 3:**
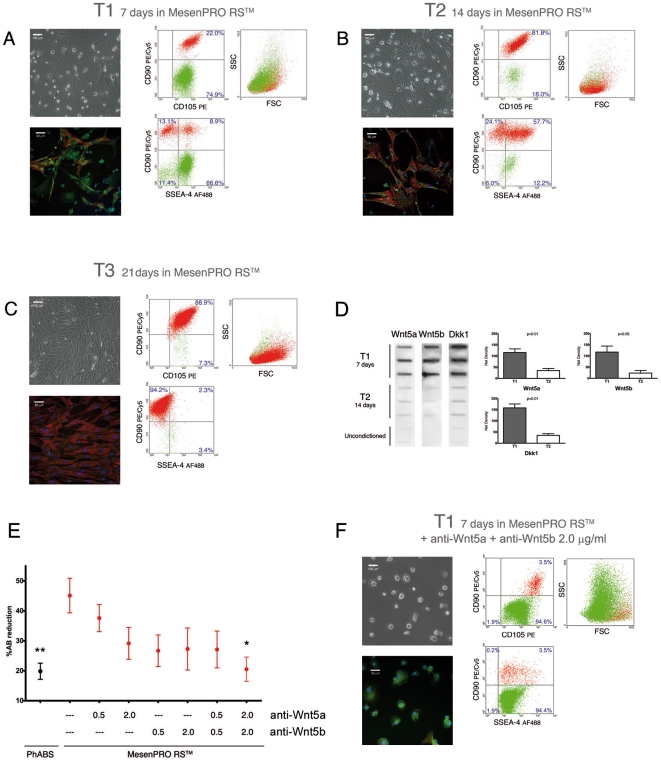
Mesenchymal differentiation of MPCs and Wnt signalling. (A) After 7 days of culture (T1) in differentiating medium some spindle-shaped cells were detectable (Leica DM-RB, HBO-50W Fluorescence 400X, merging by Leica CW4000 software). SSEA-4 (green) and CD90 (red, blue stain for DAPI) immunofluorescence and flow cytometry allowed the identification of a portion of MSCs as *early* MSCs (SSEA-4 and CD90 positive). (B) After a further 7 days (T2) cultures showed a reduced population of MPCs alongside an increased population of *early* MSCs. (C) Confluence of spindle-shaped cells was obtained after 3 weeks of differentiation (T3). Immunofluorescence showed that cells were completely negative for SSEA-4 while expressing CD90. Flow cytometry confirmed the unique phenotype of *late* MSCs (SSEA-4^neg^CD105^bright^CD90^bright^). (D) Slot-Blot of conditioned media revealed intense secretion of Wnt5a and Wnt5b at T1 and very low secretion at T2. Similarly, Dkk1 was highly secreted at T1 and reduced at T2. (E) Autocrine/paracrine actions of Wnt5a and Wnt5b were redundant and differentiation was significantly inhibited with high doses (expressed in µg/ml) of specific antibodies (* *p*<0.05, ** *p*<0.01). (F) Immunoblocking of Wnt5a and Wnt5b activity, during differentiation, resulted in the retention of MPC phenotype in about 95% of the cells, after 7 days of induction.

To investigate the role of Wnt5, MPCs were induced to differentiate to either MSCs by culturing in MesenPRO™ RS or to endothelial cells by culturing in Endo-Cult™ media. We identified two distinct phases in MPC mesenchymal differentiation: *early* and *late*. After seven days of mesenchymal induction (T1) (Figure 3A), cultures revealed a small population of flat multi-branched cells reactive to CD90 and SSEA-4. Flow cytometry confirmed the presence of two distinct cell populations on the basis of CD105 and CD90 expression. Most cells at T1 were MPCs (SSEA-4^+^CD105^dim^CD90^neg^, 76.8%±6.8, n = 3) with a minor population of MSCs (CD105^bright^CD90^bright^, 24.3%±3.6, n = 3) (Figure 3A). About 50% percent of the MSCs were SSEA-4 positive (*early* MSCs) while the remaining MSCs were SSEA-4 negative (*late* MSCs). After a further 7 days of culture (T2) (Figure 3B) MPCs were fewer than 15% (12.3%±5.6, n = 3) and most of the cells were *early* MSCs (61.3%±4.7, n = 3) with a few *late* MSCs (9.3%±2.6, n = 3). At 21 days (T3), more than 95% of the cells were *late* MSCs (SSEA-4^neg^CD105^bright^CD90^bright^, 95.3%±6.6 n = 3, Figure 3C).

At T1, intense secretion of Wnt5a (*p*<0.01 vs unconditioned media) and Wnt5b (*p*<0.01) was detected. Interestingly, at T2 Wnt5a (*p*<0.01) and Wnt5b (*p*<0.05) secretion was reduced despite the increased cellular density of mesenchymal cells. In parallel, Dkk1 secretion was higher at T1 (*p*<0.01) as compared to T2 (*p*<0.01) (Figure 3D). Mesenchymal differentiation of MPCs was completely abolished by treatment with anti-Wnt5a in combination with anti-Wnt5b (2.0 µg/ml) antibodies. The percentage of reduced Alamar Blue® was similar to unstimulated MPCs (PhABS, see materials and methods) and significantly lower when compared to MesenPRO™ RS cultures (*p*<0.05) (Figure 3E). Differentiation was not significantly inhibited when a single antibody was added to the culture, regardless of its concentration. Treatment with IgG isotype control antibody had no effect on differentiation (data not shown). These results suggest a redundant role for Wnt5a and Wnt5b in the autocrine/paracrine activation of Wnt signalling pathway, while binding of Wnt5a or Wnt5b on surface receptors was required during the induction of MPC mesenchymal differentiation (T1). Furthermore, morphological and phenotypical studies on mesenchymal differentiation cultures of MPCs (performed in presence of 2.0 µg/ml of both antibodies; anti-Wnt5a and anti-Wnt5b), confirmed that the inhibition of proliferation reported was effectively associated with a block of the mesenchymal differentiation. In fact, after 7 days of culture more than 95% of treated cells retained the expression of MPC phenotype (95.6%±4.1 n = 3, Figure 3F), alongside very low percentage of early MSCs (1.7%±1.5). Phase contrast and fluorescence microscopy revealed the almost exclusive presence of highly rifrangent rounded cells, stained brightly with anti SSEA-4 and negative to CD90 (respectively green and red in Figure 3F).

**Figure 4:**
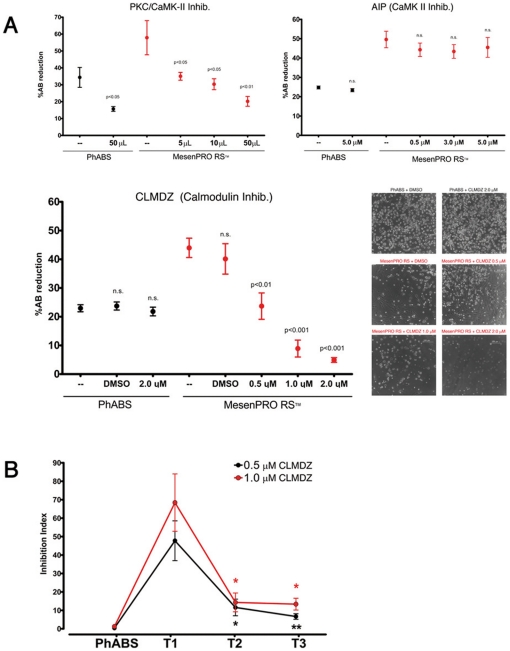
Calmodulin activation during mesenchymal differentiation of MPCs and its involvement restricted to the MPC-*early* MSC phase. (A) Treatment with inhibitors of PKC and CaMK-II resulted in a toxic effect both on differentiating (MesenPRO RS™) and resting (PhABS medium) MPCs. Specific inhibition of CaMK-II had no effects on mesenchymal differentiation while CLMDZ exerted a dose-dependent inhibiting effect. (B) The inhibition index measured in the first week of differentiation (T1) was considerably reduced when treatment with CLMDZ at 0.5 µM (ID_50_, black dots) and 1.0 µM (2×ID_50_, red dots) were performed from day 7 (T2) or 14 (T3) of differentiation (* *p*<0.05, ** *p*<0.01).

**Figure 5:**
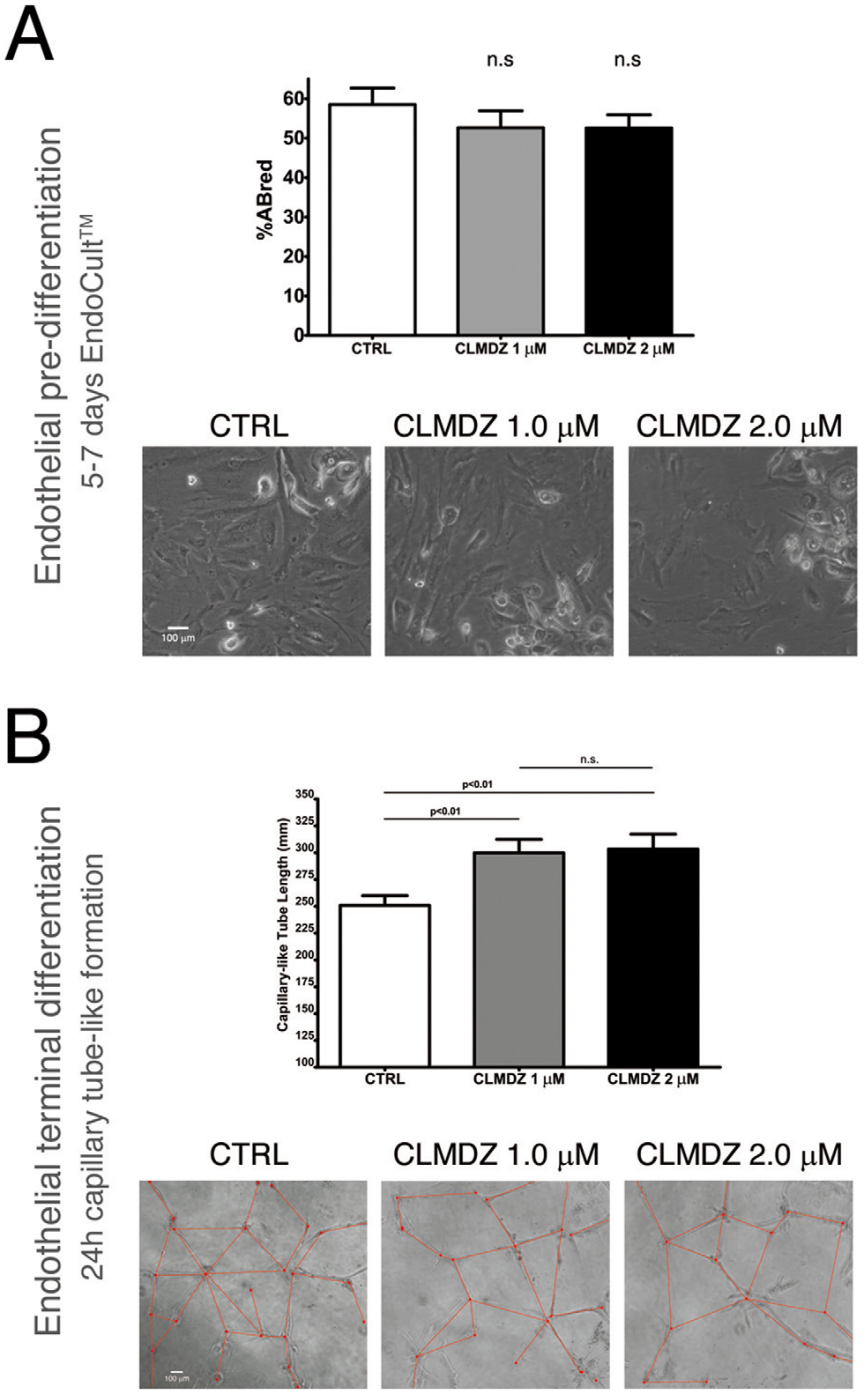
CLMDZ treatment did not affect MPC endothelial differentiation. (A) Induction toward the endothelial lineage was not inhibited by CLMDZ even at a high concentration. (B) Computer assisted measurement (24 h in Matrigel® 3D-cultures) of distances between cell bodies (red lines) revealed only a mild increase in tube length for CLMDZ treated cultures (Leica DM-IRB 100X, phase contrast, Leica QWin V3 software).

We also assayed the downstream involvement of PCK, CaMK-II, and Calmodulin. Inhibition of PKC and CaMK-II proved to be toxic both on resting (PhABS) and differentiating MPCs (MesenPRO™ RS) in a dose independent fashion (Figure 4A). On the other hand, highly specific inhibition of CaMK-II with AIP did not interfere with MPC survival or differentiation. Interestingly, blocking Calmodulin specific enzyme activity with calmidazolium chloride (CLMDZ) resulted in a dose dependent inhibition of differentiation (ID_50_ = 0.5 µM), but had no effect on resting MPCs. Cell vitality and ability to subsequently differentiate toward MSCs were conserved after removing CLMDZ (data not shown). The inhibition by CLMDZ, registered at T1 was significantly reduced at T2 and T3 (Table 1 and Figure 4B). CLMDZ was unable to inhibit MPC endothelial differentiation, even at higher concentrations (4×ID_50_), as shown by close percentages of reduced AlamarBlue® in control cultures (61.15±4.17, n = 8) vs CLMDZ at 1.0 µM (49,05±4.30, n = 8) and 2.0 µM (49,90±3.34, n = 8) (Figure 5A). Furthermore, endothelial induced cells retained the ability to form tube-like structures in Matrigel®, independently of the treatment and with mild differences in capillary-like tube length (Figure 5B). This set of data confirmed that Wnt5/Calmodulin activation is restricted to the mesenchymal lineage.

**Table 1:** Inhibition of mesenchymal differentiation by Calmidazolium Chloride (CLMDZ).

	T1	T2		T3	
CMLDZ	*Inhibition Index* (1)	*Inhibition Index*	*p value* (2)	*Inhibition Index*	*p value*
0.5 µM	47.7±10.8	9.7±4.8	0.025	6.7±1.5	0.008
1.0 µM	68.4±15.5	20.7±7.5	0.038	13.4±7.2	0.040

(1) [(%ABred_CTRL_−%ABred_Sample_)×100]/(100−%ABred_CTRL_).

(2) Significant differences to T1 for *p*<0.05, evaluated by Mann-Whitney test (n = 5).

## Discussion

We recently isolated from MSC cultures a population of MPCs that exhibited mesenchymal and endothelial differentiation potential under defined culture conditions [24]. The present report shows evidence of a multi-step model of mesenchymal differentiation. We identified a specific phenotype associated to an intermediate state between MPCs and MSCs (*early* MSCs). These cells revealed unique morphology (flat multi-branched cells) and distinctive immunophenotype (SSEA-4^+^CD105^bright^CD90^bright^), different from MPCs and from widely accepted MSCs. Higher clonogenic potential of SSEA-4^+^ MSCs had already been reported in adult mesenchymal stem cell populations [19]. We recognized SSEA-4, a marker previously thought to be specific to very early embryonic development and to hES cells, as a tracer of mesenchymal differentiation. It was highly expressed in MPCs while progressively decreasing during differentiation toward mesenchymal lineage, and it is not expressed by proliferating *late* MSCs. In parallel, cell expansion rates were very low in the first seven days of MPC differentiation. A second week of culture led to the expansion phase associated to typical MSC cultures with only a homogeneous population of *late* mature MSCs detectable.

In order to further characterize the multi-step model of MPC differentiation we investigated the possible role of Wnt signalling pathways. Our data showed that at the first step of induction *in vitro* mesenchymal differentiation of MPCs was regulated by *non-canonical* pathways through Wnt5a and Wnt5b, with a possible concomitant inhibition of Dkk-1 *canonical* pathway. Interestingly, Wnt5a and Wnt5b secretion paralleled the differentiation steps, appearing very high when the proliferation rate was low and becoming significantly reduced during the exponential cell growth.

Previous studies on MSC biology led to inconsistent results that may originate from heterogeneity in culture cell populations, characterized by asynchronous steps of differentiation. A decade ago some authors identified significant growing differences within MSC cultures for the presence of cell populations with distinct morphologies and proliferation capacities (RS1 and RS2) and described a *lag* phase and a *log* phase of growth [30], [31]. Gregory *et al.* [32] found that in the early *log* phase MSCs synthesize and secrete Dkk-1, an inhibitor of the *canonical* Wnt pathway. Proliferation of undifferentiated and pre-differentiated MSCs appeared to be predominantly regulated by the *canonical* Wnt3 pathway [10], [11], [13]. A more recent report [33] showed an increased number of CFU-F in MSC cultures exposed to Wnt3 and revealed a significant increase in population doubling time when MSCs were cultured in presence of Wnt5 and Wnt3. The Authors suggested a competitive interaction between *canonical* Wnt3a and *non-canonical* Wnt5a in mesenchymal colony formation from BMMNCs, with Wnt3a affecting self-renewal and proliferation while Wnt5a maintained MSC steady-state. In the light of our results the reported Wnt5-mediated increase in the population doubling time may be related to the recruitment of *early* MSCs, whereas Wnt3 directly stimulates the proliferation of *late* MSCs. According with this scenario, we reported apparently controversial results in standard primary MSC cultures performed using FBS-containing medium, where canonical (Wnt3) and non-canonical (Wnt5a, Wnt5b) proteins were concomitantly expressed. We hypothesize that the heterogeneity of the MSCs in these kinds of cultures [14], [16], [26], leads to the activation of different signalling pathways in different co-cultured cell populations undergoing asynchronous steps of differentiation. Conversely, the more controlled and less variable MPC differentiation protocol allows us deeply investigating the Wnt signalling activation, resolving the process in its single steps.

MPC fate could be mediated by Fzd1, which was the only Wnt receptor we found consistently expressed. Fzd1 is predicted to bind at high affinity *canonical* pathway effectors Wnt3a and β-catenin as well as *non-canonical* ligands Wnt5a, Wnt5b, and Wnt7b (Table S1). Our quantitative analysis for cytoplasmatic Wnt signalling related proteins and nuclear effectors revealed that steady state MPCs are fully equipped with the molecular machinery needed for signal transduction (Figure S1A, Text S1). They seem able to activate different signalling pathways in response to different ligands in a similar fashion to MSCs. We did not detect any expression of Wnt inhibitors in MPCs. Thus, we suggest that no specific Wnt signalling pathway is precluded to this cell population. In contrast, the activation of Wnt5-mediated mesenchymal differentiation parallels the expression of *canonical* pathway inhibitors as Dkk-1 (or presumably SFRPs) preventing activation of β-catenin in the early phase.

**Figure 6:**
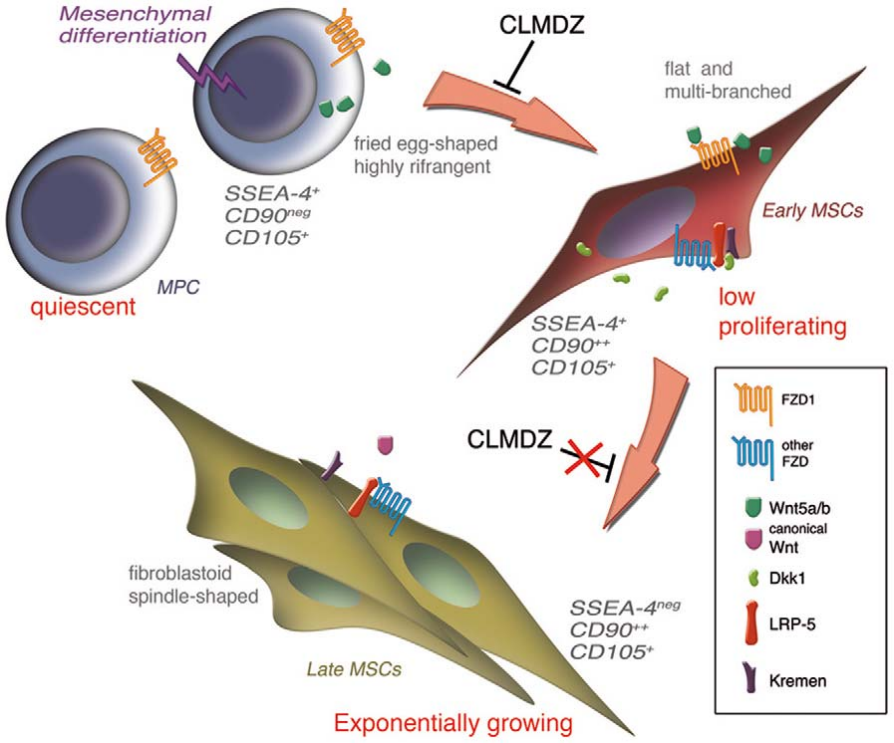
MPC mesenchymal differentiation hierarchy. Homogenous MPC populations can be induced to differentiate to *early* MSCs by activating the Wnt5/Calmodulin pathway *via* Fzd1. Terminal differentiation to *late* MSCs is related to down-regulation of either Wnt5/Calmodulin pathway or Dkk1-mediated inhibition of *canonical* signalling. Plastic adherence in different culture conditions and timing could lead to the isolation of the three different populations easily distinguishable by morphology, phenotype and proliferation rate.

As summerized in Figure 6, we identify MPCs as the hypothetical mesenchymal precursors. Activation of Wnt5/Calmodulin signalling finely tunes Fzd1 mediated induction of MPCs into a newly described intermediate differentiation stage, low proliferating *early* MSCs. Subsequent maturation to *late* MSCs (SSEA-4^neg^) gives rise to exponentially growing cultures possibly regulated by other Wnt signalling pathways.

Interestingly, under appropriate culture conditions MPCs were able to differentiate to endothelial cells giving rise to tube-like structures in Matrigel®. The inhibition of *non-canonical* Wnt5/Calmodulin pathway did not affect endothelial differentiation, thus underlining the specificity of different Wnt pathways in ruling MPC fate.

Understanding the mechanisms of MPC differentiation and self-renewal *in vitro* is crucial for future clinical applications of this promising bone marrow derived progenitor cell population. MPCs could sustain both tissue regeneration, by undergoing mesenchymal differentiation, and neo-vascolarization by undergoing endothelial differentiation.

We believe, based on the studies described above, that future studies on multipotent bone marrow stromal cells require careful revision of the widely accepted nomenclature [34], taking into account the discovery of novel phenotypes like *early* and *late* MSCs. This is easily achievable introducing routinely the SSEA-4 antigen detection into the MSC characterization panels. Moreover, we previously reported that different MSC culture conditions could lead to different percentages of co-isolated MPCs in low-passaged cells, which are not evaluated by current data analysis methods focused on CD90-positive elements. Thus, evaluation of SSEA-4^+^CD105^+^CD90^neg^ cells is also required for a complete interpretation of the results.

Lastly, we believe that many of the unresolved controversies in the field of bone marrow-derived multipotent cells could be overcame using defined number of pure MPCs and chemically defined media (MesenPRO™ RS), providing a novel and highly reproducible mesenchymal culture method, which could be useful to obtain homogeneous and synchronized cell preparations.

## Supporting Information

Text S1 **MPCs are fully equipped with the molecular machinery needed for Wnt signal transduction, as well as MSCs.**
(RTF)

Figure S1 **Quantitative RT-PCR assay for cytoplasmatic Wnt signalling related proteins and nuclear effectors.** (A) No significant difference was reported in the expression of the main phosphokinases involved in Wnt signalling between MPCs (red bars) and MSCs (black bars). (B) Only some *canonical* nuclear effectors resulted significantly more expressed on MSCs (* *p*<0.05, ** *p*<0.01).
(TIF)

Table S1 **STRING Scores of predicted protein-protein interactions.** STRING scores for predicted interactions between different Wnts (columns) and Fzd receptors, expressed at *consistent* (grey filled rows) or *mild* (unfilled rows) levels, are reported in MPCs and MSCs, respectively. Figures in bold indicate experimentally verified interactions (Source: STRING Database http://string81.embl.de).
(TIF)
