# Supplementary material for: Mesodermal Progenitor Cells (MPCs) Differentiate into Mesenchymal Stromal Cells (MSCs) by Activation of Wnt5/Calmodulin Signalling Pathway
Source: PLoS One. 2011 Sep 29;6(9):e25600. doi: 10.1371/journal.pone.0025600 (PMC3183072; doi:10.1371/journal.pone.0025600)
Supplement: Text S1 — MPCs are fully equipped with the molecular machinery needed for Wnt signal transduction, as well as MSCs. (RTF) [file pone.0025600.s001.rtf]

BONE MARROW-DERIVED MESODERMAL PROGENITOR CELLS (MPCs) DIFFERENTIATE TO MESENCHYMAL STROMAL CELLS (MSCs) BY ACTIVATION OF WNT5/CALMODULIN SIGNALING PATHWAY
Rita Fazzi, Simone Pacini, Vittoria Carnicelli, Luisa Trombi, Marina Montali, Edoardo Lazzarini, and Mario Petrini.
SUPPLEMENTAL INFORMATIONS
Quantitative RT-PCR assay did not reveal any substantial differences in the expression of intracitoplasmatic proteins involved in the Wnt signaling cascade.
Analysis of intracitoplasmatic network protein mRNAs showed no difference between MPCs and MSCs (Supplemental Fig. S1A), except for the Ras homolog gene family member U (RHOU) which was expressed by MPCs only (p<0.01). Nuclear effectors of the Wnt cascade were expressed in both cell types, although MSCs expressed higher levels of canonical associated transcription factors TCF7, LIF1, PITX2, PYGO and BCL-9 (Supplemantal Fig. S1B). Consistent expression of CCND1, CCND2, and CCND3 was evidenced in MPCs while MSCs showed down-regulation of CCND2 (p<0.05). These results confirm previously reported data [24] showing decreased c-MYC expression in MPCs as compared to MSCs (p<0.05), as well as complete silencing of FGF4 (data not shown).
