# Supplementary figures and images for: Mesodermal Progenitor Cells (MPCs) Differentiate into Mesenchymal Stromal Cells (MSCs) by Activation of Wnt5/Calmodulin Signalling Pathway
Source: PLoS One. 2011 Sep 29;6(9):e25600. doi: 10.1371/journal.pone.0025600 (PMC3183072; doi:10.1371/journal.pone.0025600)

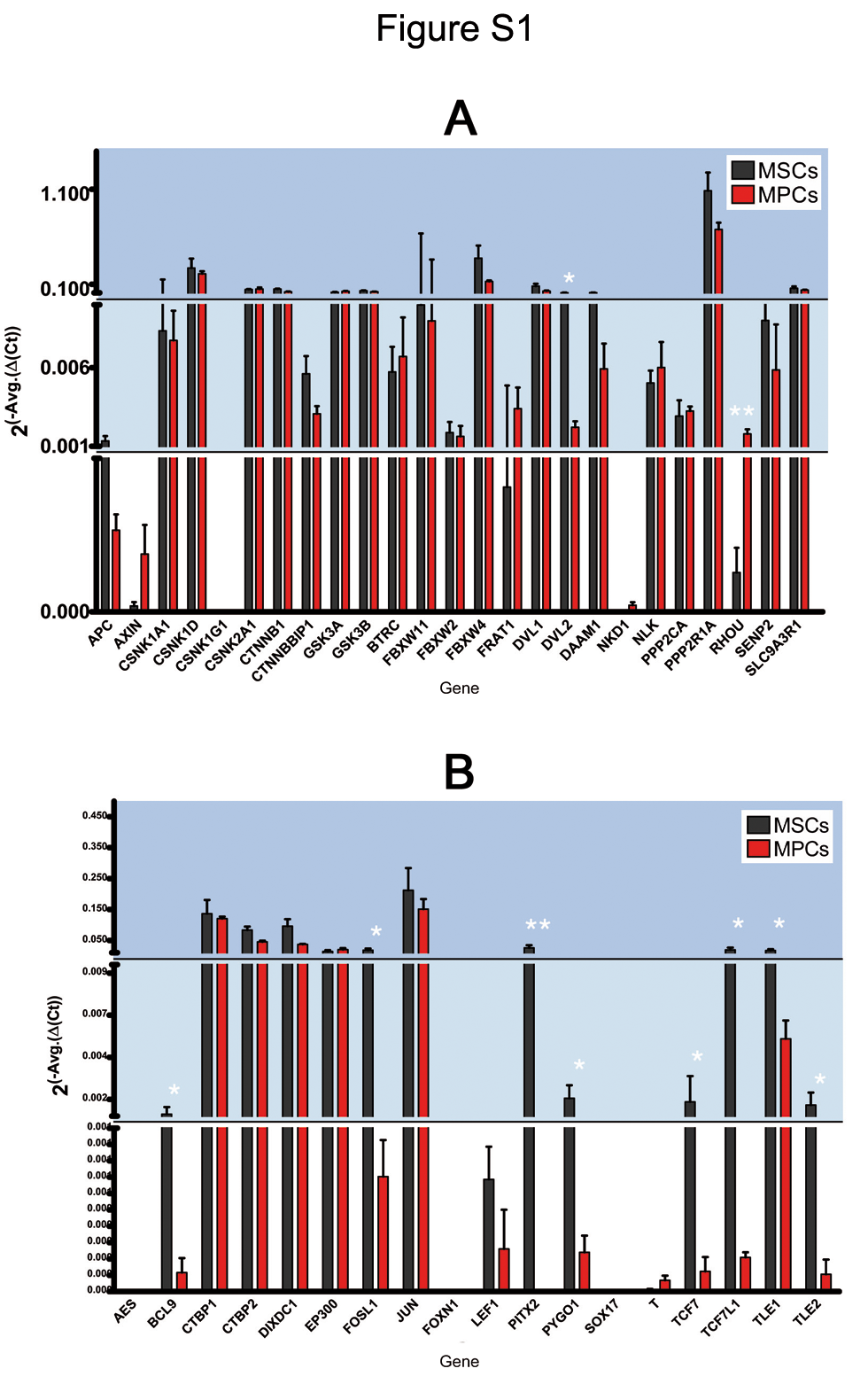

Supplement: Figure S1 — Quantitative RT-PCR assay for cytoplasmatic Wnt signalling related proteins and nuclear effectors. (A) No significant difference was reported in the expression of the main phosphokinases involved in Wnt signalling between MPCs (red bars) and MSCs (black bars). (B) Only some canonical nuclear effectors resulted significantly more expressed on MSCs (* p<0.05, ** p<0.01). (TIF) [file pone.0025600.s002.tif]

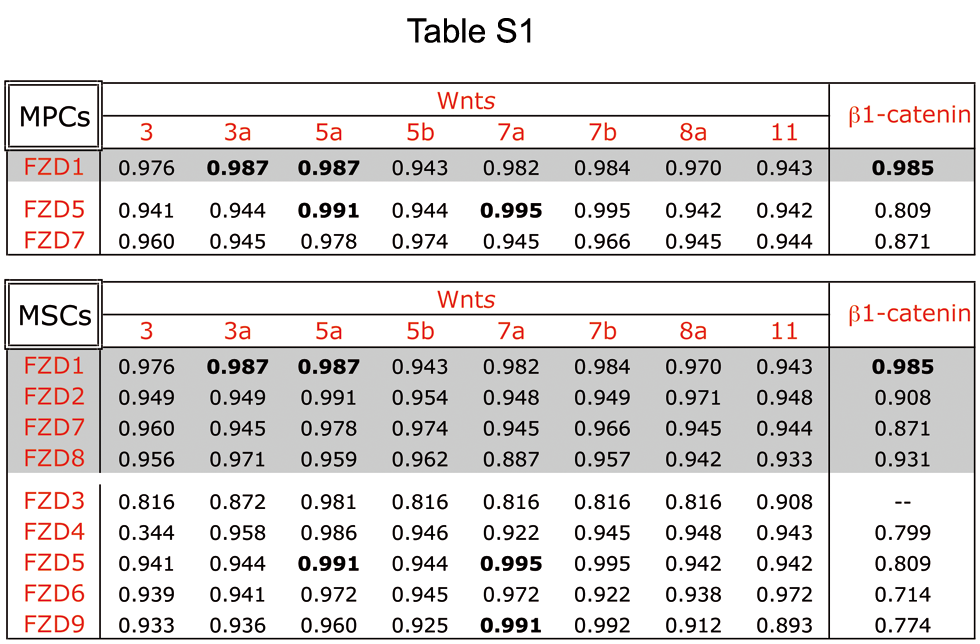

Supplement: Table S1 — STRING Scores of predicted protein-protein interactions. STRING scores for predicted interactions between different Wnts (columns) and Fzd receptors, expressed at consistent (grey filled rows) or mild (unfilled rows) levels, are reported in MPCs and MSCs, respectively. Figures in bold indicate experimentally verified interactions (Source: STRING Database http://string81.embl.de). (TIF) [file pone.0025600.s003.tif]
